# Biodegradable and Biocompatible Biomaterial, Polyhydroxybutyrate, Produced by an Indigenous *Vibrio* sp. BM-1 Isolated from Marine Environment

**DOI:** 10.3390/md9040615

**Published:** 2011-04-18

**Authors:** Yu-Hong Wei, Wei-Chuan Chen, Ho-Shing Wu, Om-Murugan Janarthanan

**Affiliations:** 1 Graduate School of Biotechnology and Bioengineering, Yuan Ze University, Chung-Li, Taoyuan 320, Taiwan; E-Mails: itispay@gmail.com (W.-C.C.); murugan_jana@yahoo.co.in (O.-M.J.); 2 Department of Chemical Engineering and Material Science, Yuan Ze University, Chung-Li, Taoyuan 320, Taiwan; E-Mail: cehswu@saturn.yzu.edu.tw

**Keywords:** polyhydroxybutyrate, biomaterial, marine microorganisms, *Vibrio* sp. BM-1

## Abstract

Polyhydroxybutyrate (PHB) is one of the polyhydroxyalkanoates (PHAs) which has biodegradable and biocompatible properties. They are adopted in the biomedical field, in, for example, medical implants and drug delivery carriers. This study seeks to promote the production of PHB by *Vibrio* sp. BM-1, isolated from a marine environment by improving constituents of medium and implementing an appropriate fermentation strategy. This study successfully developed a glycerol-yeast extract-tryptone (GYT) medium that can facilitate the growth of *Vibrio* sp. BM-1 and lead to the production of 1.4 g/L PHB at 20 h cultivation. This study also shows that 1.57 g/L PHB concentration and 16% PHB content were achieved, respectively, when *Vibrio* sp. BM-1 was cultivated with MS-GYT medium (mineral salts-supplemented GYT medium) for 12 h. Both cell dry weight (CDW) and residual CDW remained constant at around 8.2 g/L and 8.0 g/L after the 12 h of cultivation, until the end of the experiment. However, both 16% of PHB content and 1.57 g/L of PHB production decreased rapidly to 3% and 0.25 g/L, respectively from 12 h of cultivation to 40 h of cultivation. The results suggest that the secretion of PHB depolymerase that might be caused by the addition of mineral salts reduced PHB after 12 h of cultivation. However, work will be done to explain the effect of adding mineral salts on the production of PHB by *Vibrio* sp. BM-1 in the near future.

## 1. Introduction

Polyhydroxyalkanoates (PHAs) are biodegradable materials, which are accumulated to store carbon and energy in various microorganisms [1–3]. Their accumulation is limited by nutritional factors, such as nutrient deficiency or the presence of excess carbon [2,3]. PHAs are classified by the number of carbon atoms in their monomers. In “short-chain length” PHAs such as polyhydroxybutyrate (PHB) and polyhydroxyvalerate, the number of carbon atoms in the monomer is three to five. In “medium chain-length” PHAs, the number of carbons in the monomers range from six to sixteen. PHAs have the potential to replace petroleum-based plastics as biomedical materials for use in surgical pins, sutures, staples, blood vessel replacements, bone replacements and plates, medical implants and drug delivery devices owing to their superior biodegradability and biocompatibility. PHB is the most commonly used PHA and the metabolic pathways of PHB have been elucidated in detail [4]. The properties of PHB are similar to those of various synthetic thermoplastics such as polypropylene. Various microorganisms completely degrade PHB to water and carbon dioxide under aerobic conditions and to methane under anaerobic conditions.

Polyhydroxyalkanoates (PHAs) are polyesters that are synthesized by various microorganisms such as *Cupriavidus necator*, *Alcaligenes latus*, *Aeromonas hydrophila*, *Pseudomonas putida* and *Bacillus* spp. [1–4]. Several halophilic microbes (including *Haloferax mediterranei*, *Vibrio* spp., *V. natriegens*, *V. nereis* and *V. harveyi*) reportedly produce PHB [5–7]. The identification of the gene that is involved in PHA synthesis, polyhydroxyalkanoic acid synthase, was verified using *V. parahemolyticus* and *V. cholera*. The benefits of halophilic microbes in PHA production are that they produce PHA with high molecular weight, such as P(3HB-*co*-3HV). Therefore, they have potential industrial applications and may reduce the cost by some fermentation strategies, such as the immobilization of NaCl onto the walls of bioreactors [8–10]. However, the disadvantage of halophilic microbes in PHA production is their low productivity [8–10]. Few reports on marine PHA-producing microorganisms have been published, although *Vibrio* spp. A gram negative bacterium has been isolated from a marine environment in the north of Taiwan, and was identified as a *Vibrio* sp. BM-1 by the phylogenic analysis of its 16S rDNA. Hence, the production of PHB by marine microorganism *Vibrio* sp. BM-1 may be developed industrially.

Mineral salts such as Na_2_HPO_4_, KH_2_PO_4_ and MgSO_4_·7H_2_O are the important for supporting bacterial life and as the critical material for synthesizing metabolites [11,12]. Accordingly, in this study, the effects of mineral salts on PHB production were further demonstrated by gas chromatography (GC) analysis.

## 2. Results and Discussions

### 2.1. Effect of Carbon Source on PHB Production

In this investigation, the effects of carbon sources (glucose, fructose, starch, molasses, sucrose, mannitol, glycerol and acetic acid), at a fixed concentration of 10 g/L, on the production of PHB by marine microorganism *Vibrio* sp. BM-1, were evaluated. Of the various carbon sources, glycerol positively affected PHB production with a concentration of 0.58 g/L when it was the sole carbon source (Table 1). Moreover, *Vibrio* sp. BM-1 was able to take up some of the other carbon sources (*i.e.*, starch, fructose and mannitol) as nutrients for producing PHB. However, low PHB production and low cell growth were observed when the carbon source was sucrose, molasses or starch. Glucose, Fructose, acetic acid and mannitol as nutrient sources for *Vibrio* sp. BM-1 yielded a high PHB production, PHB content and cell dry weight (CDW). *Vibrio* sp. BM-1 adapted used glycerol as a carbon source, resulting in the greater PHB production than obtained using other carbon sources. Therefore, glycerol as the carbon and the glycerol-yeast extract-tryptone (GYT) medium were used hereinafter.

### 2.2. Effect of Various Concentrations of Complex Nitrogen Sources on PHB Production

**Figure 1.**
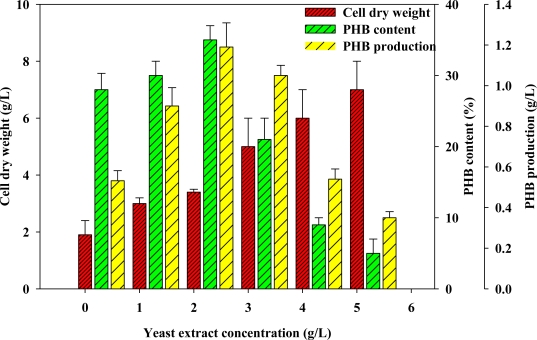
Analyses of effect of yeast extract concentration on CDW, PHB content and PHB production.

The GYT medium contained two complex nitrogen sources—yeast extract and tryptone. Both forms of nitrogen were fixed at various concentrations to evaluate the optimal concentration for PHB production. The variation of cell growth rate with initial yeast extract concentration was similar to that of PHB content at low yeast extract concentration (<2 g/L) (Figure 1). As shown in Figure 1, the cell concentration increased with yeast extract concentration from 0 to 2 g/L. Likewise, as also indicated in Figure 1, the PHB content produced by the batch-cultivated *Vibrio* sp. BM-1 also increased considerably with yeast extract concentration, eventually reaching its highest value of 34.79% at a yeast extract concentration of 2 g/L. However, further increasing the yeast extract concentration did not elevate the PHB content, which essentially leveled off for yeast extract concentrations of more than 2 g/L, with a plateau value of around 34.79% (Figure 1).

The literature reveals that the proper yeast extract concentration is a common nutritional requirement for the cultivation of most microorganisms [13]. Yeast extract contains amino acids such as glycine and betaine. Theoretically, yeast extract positively affects the growth of microorganisms and their production of PHB. However, the consumption of excessive yeast extract by microorganisms can cause them to grow rather than produce PHB [14]. Indeed, an excess of yeast extract can hinder the production of PHB by microorganisms [14]. Accordingly, based on the results in this investigation, the optimal yeast extract concentration was 2 g/L. In this study, the uses of a yeast extract-containing medium increased cell concentration and PHB production by *Vibrio* sp. BM-1. More research must be performed to clarify the role of yeast extract in the fermentation of PHB by the *Vibrio* sp. BM-1.

**Figure 2.**
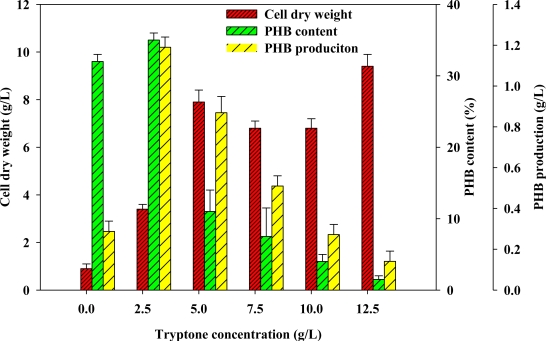
Analyses of effect of tryptone concentration on CDW, PHB content and PHB production.

The effects of concentration of another nutrient source, tryptone, on PHB production were also evaluated. Figure 2 demonstrates that PHB production depended on the tryptone concentration. The results indicate that PHB production and the PHB content decreased as tryptone concentration increased by comparison with a control group, for which the concentration of tryptone was 2.5 g/L. Consistent with the experimental results, tryptone may have a similar effect to yeast extract on PHB production when in excess. Therefore, the optimal tryptone concentration of 2.5 g/L maximized PHB production, PHB content and CDW (Figure 2). Accordingly, the GYT medium contained 2 g/L of yeast extract and 2.5 g/L of tryptone.

### 2.3. Effect of Concentration of Sodium Chloride on PHB Production

**Figure 3.**
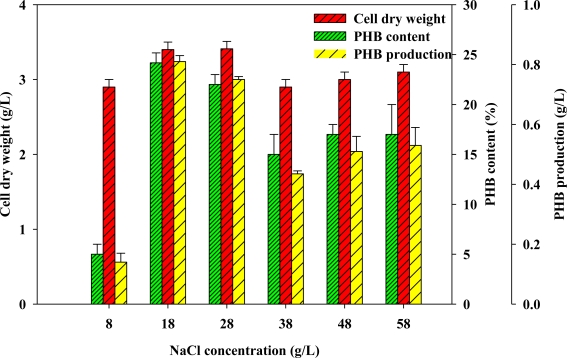
Analyses of effect of NaCl concentration on CDW, PHB content and PHB production.

The NaCl concentration in the medium may participate crucially in PHB production since *Vibrio* sp. BM-1 is a marine microorganisms [15]. An attempt was made to elucidate the most effective strategies for NaCl supplementation to optimize PHB production. *Vibrio* sp. BM-1 was grown using GYT medium to which was added various concentrations of NaCl (0 g/L, 8 g/L, 18 g/L, 28 g/L, 38 g/L, 48 g/L and 58 g/L) to examine the effect of NaCl concentration on PHB production. Figure 3 reveals that PHB production depends on NaCl concentration. The maximal PHB production increased with NaCl concentration up to 18 g/L, but seriously decreased as the NaCl concentration was further increased from 18 g/L to 58 g/L. Therefore, 18 g/L appeared to be the optimal concentration of NaCl, yielding a maximum PHB production of 0.81 g/L, a PHB content of 24.17% and a CDW of 3.37 g/L—all greater than obtained using the NaCl-free GYT medium. However, poor cell growth and low PHB concentration were observed when the NaCl concentration exceeded 18 g/L (Figure 3). This finding reflects the necessity of controlling the salinity of the culture within a proper range to prevent high osmotic stress, and its effect on PHB production.

### 2.4. Effect of Mineral Salts on PHB Production

**Figure 4.**
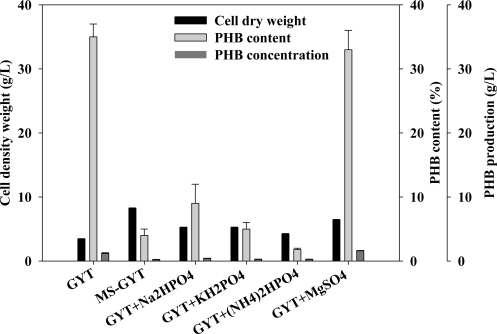
Analyses of effect of various added mineral salts on CDW, PHB content and PHB production.

Adding mineral salts individually to the GYT medium had interesting effects on PHB production (Figure 4). The GYT medium plus one mineral salt, Na_2_HPO_4_, KH_2_PO_4_, (NH_4_)_2_HPO_4_ or MgSO_4_·7H_2_O reduced PHB production. Figure 4 shows that PHB production in MS-GYT medium was lower than that in GYT medium. The MS-GYT medium (GYT medium plus four mineral salts) yielded one sixth of the PHB production (from 1.2 g/L to 0.2 g/L) that was obtained in GYT medium, but increased CDW (from 3.4 g/L to 8.2 g/L), as did the GYT medium with one added mineral salt. The results suggest that mineral salts inhibited PHB production by *Vibrio* sp. BM-1, but not inhibited cell growth (Figure 4).

**Figure 5.**
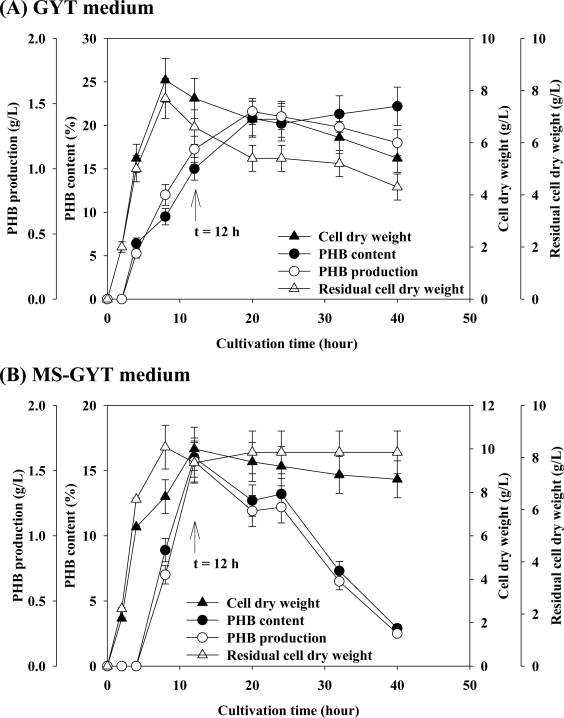
Profile of PHB production, PHB content, CDW and residual CDW, when *Vibrio* sp. BM-1 is cultivated with: (**A**) GYT medium; and (**B**) MS-GYT medium. The arrow at *t* = 12 h indicated that after 12 h cultivation, *Vibrio* sp. BM-1 with GYT or MS-GYT medium started gradually to decrease PHB accumulation due to bacterial death (CDW from 8.4 g/L to 5.4 g/L, Figure 5A) or consumed their own PHB for maintaining growth (CDW maintained at around 8.2 g/L, Figure 5B).

Figure 5A demonstrates that the PHB content and PHB production elevated at 15% and 1.15 g/L respectively, with increasing CDW from 0 g/L to 7.7 g/L when *Vibrio* sp. BM-1 was cultivated with GYT medium for 12 h, and remained at a constant level (around 20% and 1.2 g/L) until the end of the experiments. The CDW and residual CDW followed similar trends to that of the PHB production. These results suggest that the cultivation environment in the stationary phase was not conductive for bacterial growth, resulting in the death of the bacteria from 8.4 g/L to 5.4 g/L when *Vibrio* sp. BM-1 was cultivated with GYT medium. Figure 5B also reveals that the PHB production and PHB content elevated from 0 g/L to 1.57 g/L and 0% to 16%, respectively, when *Vibrio* sp. BM-1 was cultivated with MS-GYT medium for 12 h. Both CDW and residual CDW remained constant at around 8.2 g/L after the 12 h of cultivation, until the end of the experiment. However, both 16% of PHB content and 1.57 g/L of PHB production decreased rapidly to 3% and 0.25 g/L, respectively from 12 h of cultivation to 40 h of cultivation. These results concerning cultivation with MS-GYT medium suggest that the decrease in the production of PHB was caused by both the cultivation environment and intracellular PHB depolymerase [16,17]. The abdominal cultivation environment caused *Vibrio* sp. BM-1 to degrade PHB for maintaining cellular growth, and not to store energy sources in the stationary phase when the cultivation medium contained mineral salts. Besides, the lack of nutrients in the cultivation environment also reduced the PHB content of *Vibrio* sp. BM-1 that is used for cell growth.

## 3. Experimental Section

### 3.1. Microorganism and Culture Medium

A gram negative bacterium was isolated from a marine environment in northern Taiwan and was identified as a *Vibrio* sp. BM-1 by phylogenic analysis of the 16S rDNA [11]. Two media, GYT and MS-GYT, were used to cultivate *Vibrio* sp. BM-1 and thereby accumulate PHB. The GYT medium consisted of glycerol (10 g/L), yeast extract (2 g/L), tryptone (2.5 g/L) and NaCl (18 g/L). The culture medium MS-GYT was also used to synthesize PHB but was produced by adding mineral salts, including Na_2_HPO_4_ (3.7 g/L), KH_2_PO_4_ (1 g/L), (NH_4_)_2_HPO_4_ (0.5 g/L) and MgSO_4_·7H_2_O (0.2 g/L), to the GYT medium. The addition of mineral salts to the GYT medium had interesting effects on PHB synthesis.

### 3.2. Culture Condition

Before inoculation, the *Vibrio* sp. BM-1 was incubated for 12 h at 30 °C in a 250 mL Erlenmeyer flask that contained 50 mL of preculture medium (GYT), and was spun at 200 rpm under aerobic conditions. Various carbon-sources were selected to evaluate their effect on PHB production. The carbon sources were glucose, fructose, sucrose, molasses, mannitol, starch acetic acid and glycerol. All the effects of the concentrations of carbon sources, complex nitrogen sources and NaCl on PHB production in the batch culture were examined at 30 °C and an agitation speed of 200 rpm.

### 3.3. Quantifications of Cell Growth and PHB

Cell growth was monitored by measuring turbidity at an optical density 600 nm. Cell concentration, defined as cell dry weight (CDW) per liter of culture broth, and PHB concentration were determined as described elsewhere [18]. Residual glycerol concentrations were estimated using an HPLC column (Aminex HPX-87H, BIO-RAD), mobile phase: 5 mM H_2_SO_4_, flow rate: 0.5 mL/min, RI detector: L-2490 (Hitachi, Tokyo, Japan).

### 3.4. Analytical Methods

The GC analysis was performed after methanolyzing the polymer in sulfuric acid and methanol as described by previous studies [19,20]. Ten milligrams of biomass was added to 1 mL chloroform and 1 mL acidic methanol (2.8 M H_2_SO_4_ in methanol); 1 g/L benzoic acid was used as an internal standard. Methanolysis was performed at 100 °C for 2 h. The PHA content and composition were determined by gas chromatography (Focus GC, Thermo, U.S.). After vigorous shaking, 1 mL of distilled water was added by pipetman of 1 mL. A 0.2 μL underlayer was injected into the gas chromatograph. The initial GC column temperature was 80 °C, which was held for 1 min, before being increased to 250 °C at a rate of 20 °C/min, which was maintained for 1.5 min.

## 4. Conclusions

This study proved that GYT medium was the most effective medium for cultivating *Vibrio* sp. BM-1. PHB production, PHB content and CDW were increased from 0.58 g/L to 0.81 g/L, 15.68% to 24.17% and 0.58 g/L to 3.37 g/L, respectively, when the GYT medium was used as a cultivation medium. Mineral salts such as Na_2_HPO_4_, KH_2_PO_4_, (NH_4_)_2_HPO_4_ and MgSO_4_·7H_2_O significantly affected PHB production. The results suggest that the secretion of PHB depolymerase that might be caused by the addition of mineral salts reduced PHB after 12 h of cultivation. However, the effects of mineral salt which resulted in declining PHB production must be investigated further. The findings of this study provide a reference for further research into the use of PHB to manufacture biodegradable polymers using marine microorganisms.

## Figures and Tables

**Table 1.** Effect of various carbon sources on cell dry weight (CDW), Polyhydroxybutyrate (PHB) content and PHB production.

**Carbon Sources**	**CDW (g/L)**	**PHB Content (%)**	**PHB Produciton (g/L)**
Glucose	2.73 ±0.03	17.49 ±0.18	0.35 ±0.03
Fructose	2.78 ±0.03	17.92 ±2.11	0.52 ±0.05
Sucrose	0.76 ±0.01	*ND.	*ND.
Molasses	0.69 ±0.01	2.39 ±0.23	0.02 ±0.01
Starch	0.54 ±0.05	*ND.	*ND.
Acetic acid	5.04 ±0.05	4.99 ±0.67	0.25 ±0.04
Mannitol	3.06 ±0.03	10.77 ±1.12	0.33 ±0.02
Glycerol	3.69 ±0.04	15.68 ±1.73	0.58 ±0.05

* ND.: Not detected.
